# Autecology of an oscillating population of a novel host-associated *Phaeobacter* species proliferating in marine bryozoans

**DOI:** 10.1093/ismeco/ycaf178

**Published:** 2025-10-09

**Authors:** Mikkel Bentzon-Tilia, Nathalie N S E Henriksen, Morten D Schostag, Aaron J C Andersen, Jette Melchiorsen, Mikael L Strube, Lone Gram

**Affiliations:** Department of Biotechnology and Biomedicine, Technical University of Denmark, Søltofts Plads Bldg. 221, 2800 Kgs. Lyngby, Denmark; Department of Biotechnology and Biomedicine, Technical University of Denmark, Søltofts Plads Bldg. 221, 2800 Kgs. Lyngby, Denmark; Department of Biotechnology and Biomedicine, Technical University of Denmark, Søltofts Plads Bldg. 221, 2800 Kgs. Lyngby, Denmark; Department of Biotechnology and Biomedicine, Technical University of Denmark, Søltofts Plads Bldg. 221, 2800 Kgs. Lyngby, Denmark; Department of Biotechnology and Biomedicine, Technical University of Denmark, Søltofts Plads Bldg. 221, 2800 Kgs. Lyngby, Denmark; Department of Biotechnology and Biomedicine, Technical University of Denmark, Søltofts Plads Bldg. 221, 2800 Kgs. Lyngby, Denmark; Department of Biotechnology and Biomedicine, Technical University of Denmark, Søltofts Plads Bldg. 221, 2800 Kgs. Lyngby, Denmark

**Keywords:** *Phaeobacter*, roseobacters, tropodithietic acid, TDA, roseobacticides, bryozoans

## Abstract

*Phaeobacter* are marine alphaprotebacteria capable of producing a potent antibacterial compound, tropodithietic acid. Here we demonstrate that they are part of the microbiome of marine bryozoans where they during warmer months reach 10^5^ CFU/g. The levels exhibited a bimodal fluctuation, in both bryozoans and seawater across seasons. However, the population of *Phaeobacter* sp. was already established in the bryozoans prior to the peak in seawater and did not accumulate as a function of filter feeding on phytoplankton biomass, suggesting that the seawater population is likely seeded from the bryozoan-associated *Phaeobacter* sp. population rather than the opposite. By comparing whole-genome sequences of more than 100 bryozoan-associated *Phaeobacter* isolates sampled over a 12-year period, we found that all belonged to the same novel species and no systematic genetic changes occurred within it over the 12 year sampling period despite the fact that the population oscillated from below the limit of detection and across five orders of magnitude to 5.2 Log_10_ CFU g^−1^ bryozoan within individual years and hence were subject to drift. All isolates had the genetic capacity to produce tropodithietic acid (TDA) and the algicidal compounds, roseobacticides. The genes encoding the enzymes for TDA biosynthesis remained stable over time, indicating a conserved phenotype important in the ecophysiology of the bacteria. TDA biosynthetic genes were actively transcribed within the bryozoan host further corroborating the notion that the secondary metabolites of this novel host-associated *Phaeobacter* sp. may be central to its role within the bryozoan microbiome.

## Introduction

Coastal and inland aquatic systems, such as estuaries, transport organic material, and inorganic nutrients between land and sea. While such waters cover a small fraction of the surface area of Earth, they play a disproportionately large role in global carbon cycling [1]. The high productivity in these systems sustain a wealth of marine animals, including filter feeders such as mussels and moss animals (bryozoans), which form high biomass aggregations generating local hotspots of biogeochemical cycling by concentrating nutrients [2]. The turnover of organic matter in bryozoans likely involves associated symbiotic microorganism [3], however, while bryozoans, together with sponges and cnidarians, are among the dominant epibiotic groups in marine systems, information about their associated microbiome remains scant relative to other metazoans [4, 5].

Members of the alpha-proteobacterial *Roseobacter* group, or “roseobacters” [6], are ubiquitous in marine systems accounting for an average of 3.8% of bacterioplankton cells in seawater globally [7]. They likely play a key role in the remineralization of organic matter and in the marine sulfur cycle due to their capacity to utilize algal dimethylsulfonioproprionate (DMSP) in their primary metabolism [8, 9]. Some roseobacters produce the sulfur-containing tropone derivative tropodithietic acid (TDA), a secondary metabolite, which at high concentrations have broad-spectrum antimicrobial activity, while it may serve other roles in an environmental context, including iron chelation and signaling [10]. The antimicrobial mode of action of TDA has been investigated in *Escherichia coli*, where it likely exerts its activity by disrupting the proton motive force by binding a metal ion in the cytosol and exchanging it for an extracellular proton prior to its reentry into the target cell [11]. In the marine pathogen *Vibrio vulnificus*, sublethal concentrations of TDA induce responses related to oxidative stress, suggesting a similar mode of action in vibrios [12] and potentially other fast growing competitors. While TDA has broad-spectrum activity [13], the natural microbiomes in which TDA-producers live are usually not affected by the introduction of TDA-producers, suggesting subtle and specific effects *in situ* [14–19] or the presence of widespread, yet unknown tolerance mechanisms, in co-occurring community members.

Several species across three widespread roseobacter genera, *Tritonibacter* (formerly *Epibacterium*, *Ruegeria*, or *Silicibacter*), *Pseudovibrio*, and *Phaeobacter* (formerly *Roseobacter*), carry the capacity to produce TDA [20–24], suggesting a central role of this metabolite in the ecology of these species. While *Tritonibacter* spp. are believed to be the predominant open-ocean TDA producers (0.2% relative abundance; [25]), *Phaeobacter* spp. represent a smaller fraction of the global ocean microbiome (0.03%; [25]) and are commonly found living on surfaces or in symbioses with eukaryotic host organisms [26]. In particular, a connection between microalgae and roseobacters, including *Phaeobacter* spp., has been suggested due to a positive correlation with chlorophyll *a* concentration [7]. This connection is reinforced by the observation that relative abundances of roseobacters collectively can reach up to 30% of bacterial populations during microalgal blooms [8, 27]. The underlying mechanism of *Phaeobacter*-algal symbiosis likely involves TDA as a potential killer of algal pathogens as well as additional host- and bacterial derived metabolites, e.g. the phytohormone indole-3-acetic acid and the roseobacticides, which are algacidal metabolites sharing a number of biosynthetic enzymes with TDA [28] that are released by the bacterium upon bloom senescence [29, 30]. Other secondary metabolites connected to TDA, such as the TDA analog methyl-troposulfenin are produced by certain *Phaeobacter* species, yet the natural roles of these analogs remain obscure.

*Phaeobacter* spp. have repeatedly been isolated from live feeds in aquaculture [22, 31–33] and a few studies have detected strains of the genus in harbor environments, including from surface attached bryozoans [34, 35]. However, it remains unknown whether *Phaeobacter* constitutes a permanent member of these communities and if the capability to produce TDA is permanent phenotype or evolves over time.

To understand the ecological roles and niche differentiation of *Phaeobacter*, it is essential to investigate the spatiotemporal dynamics of the population. Such dynamics are influenced by ecological factors, including resource availability, predation by protozoans, viruses, and the abundance of potential host organisms [36]. Since other *Phaeobacter* spp. are known to interact and co-exist with algae, their population dynamics may be influenced by the seasonality of microalgae found in temperate waters. Additionally, investigating the temporal patterns of *Phaeobacter* spp. will help determine the timing and conditions under which they produce TDA and roseobacticides, providing insight into their interactions with other microorganisms, the environment, and their broader ecological impact.

The purpose of the present study was to uncover the autecology of roseobacters associated with an estuarine harbor environment. Specifically, we examined their abundance patterns across multiple temporal scales (ranging from months to years) to assess whether, and how, population diversity varied across time. Given that TDA production is conserved in *Phaeobacter* species isolated from diverging niches, we also set out to evaluate the long-term conservation of this trait by comparing TDA biosynthetic gene clusters (BGCs) from isolates collected over a decade. Unraveling taxonomic and functional changes over a more than 10-year sampling scale can form the basis for understanding the ecology and potential role of these organisms.

## Materials and methods

### Sampling and metadata collection

A total of 20 samplings were conducted between April 2009 and September 2021 in Jyllinge harbor in Roskilde Fjord (55° 45′ 30.35" N, 12° 06' 44.17" E). Procedures for samples collected in April–August 2009 and November 2009–June 2010 are described in [37], and for samples collected in October 2009, August 2011–September 2012 in [34]. Sampling procedures for the remaining samples are described in the following.

Triplicate seawater samples were collected in autoclaved 1 l Schott bottles and ~10 g of surface attached bryozoan biomass was collected directly in triplicate 50 ml falcon tubes. In situ measurements of temperature, salinity and O_2_ concentrations were done using a handheld Professional Plus instrument (YS6050000; YSI, Yellow Springs, OH). pH was determined for the triplicate water samples using a PHM83 ULTRACAL pH-meter (Radiometer, Copenhagen, Denmark). Chlorophyll *a* (Chl *a*) concentrations were obtained from a continuous monitoring program run by the Danish Nature Agency.

### Quantification of cultivable bacteria, isolation, and whole genome sequencing of *Phaeobacter* sp.

Total abundances of cultivable bacteria were determined by serial dilution and CFU counts on Marine Agar (MA; Difco 2216) and for TDA-producing *Phaeobacter* sp. as distinct brown colored colonies leaving brown pigmentation (the TDA-iron complex) in the agar upon removal [38]. For seawater, tenfold serial dilutions in 2% Instant Ocean (IO; Aquarium Systems Inc., Sarrebourg, France) were plated directly on MA, and for bryozoan samples, 2 g of biomass was homogenized in 18 ml of 2% IO using a T25 ULTRA-TURRAX® disperser (IKA, Staufen, Germany) prior to serial dilution and plating. The significance of differences in abundance was determined using a Kruskal–Wallis test followed by a Dunn’s multiple comparisons test. The reported *P*-values are adjusted for multiple testing using the Bonferroni correction.

Colonies exhibiting distinct diffusing brown coloration were collected whenever observed on MA plates (11 out of 19 samplings), streaked to purity, and stored at −80°C. The cultures were revived on MA and individual colonies were used to inoculate 5 ml of MB, which was subsequently incubated at 25°C for 24 h and genomic DNA was extracted using the Maxwell® 16 LEV Blood DNA Kit (Promega, Madison, WI, USA) and processed with the Maxwell® 16 instrument according to instructions provided by the manufacturer. DNA was eluted in 10 mM Tris–HCl, pH 8.5. gDNA concentrations and purity were determined using the Qubit 2.0™ fluorometer (Invitrogen) and the DS-11+ nanodrop spectrophotometer (DeNovix), respectively. Paired-end (2 × 150 PE) sequencing was performed by Novogene on the Illumina NovaSeq 6000 platform. The reads were assembled with SPAdes 3.13.0 through the Shovill pipeline [39, 40], after which the Average Nucleotide Identity (ANI) between isolates and reference strains (*Phaeobacter inhibens* 2.10, *P. inhibens* DSM 16374, and *Phaeobacter piscinae* DSM 103509^T^) were calculated by PYANI using the MUMmer algorithm [41, 42]. Hierarchical clustering of the resulting ANI-matrix suggested a division of the isolates into eight distinct clades, and from each of these a representative member was uploaded to the Type Strain Genome Server [43] to corroborate the novelty of the species.

### Conservation of tropodithietic acid genes

To investigate if genes for secondary metabolism were subjected to increased evolutionary pressure relative to the core genome, the genes of the TDA cluster (*tdaA-F*) were compared to the housekeeping genes *gyrA*, *rpoB,* and *recA*. Briefly, the protein products of each gene were aligned, and the Shannon entropy of each amino acid position was calculated. These entropies were then summed and standardized by the length of the protein, giving a single value for the conservation of each gene. Significance of the scale of TDA-gene entropies vs housekeeping entropies was calculated with a T-test.

### Metabolomics

To determine if the isolates represented different chemotypes, 11 isolates representing eight subspecies, or “clades”, identified by genome comparison, and different years of isolation were chosen. The 11 representatives were analyzed using ultrahigh-performance liquid chromatography coupled to high resolution electrospray ionization mass spectrometry (UHPLC–MS). Strains were grown on Marine Agar (MA; Difco2216) for 24 h at 25°C and triplicate colonies of each strain were inoculated into Marine Broth (MB; Difco2216) and incubated for 24 h at 25°C, shaking at 250 rpm. 20 *µ*l of each of the MB cultures were spotted onto MA plates, which were subsequently incubated at 25°C for 48 h. Individual spots including the underlying agar were transferred to microcentrifuge tubes and extracted with 1 ml of isopropanol:ethyl acetate (1,3, v/v) containing 1% formic acid under ultrasonication for 60 min. Subsequently, the liquid phase was evaporated to dryness under N_2_ and re-dissolved in 200 μl methanol. A negative medium control and biological triplicates of the three reference strains *P. inhibens* DSM 17395, *P. inhibens* 2.10, and *P. piscinae* DSM 103509^T^ were included as well.

Chromatographic separation of the extracts was achieved on an Agilent 1290 UHPLC using a Poroshell 120 phenyl-hexyl (150 × 2.1 mm i.d., 1.9 μM) stationary phase from Agilent Technologies. The stationary phase was held at a constant 40°C with metabolites eluted using a linear gradient at a constant flow rate of 350 μl min^−1^. The gradient utilized consisted of two eluents (Eluent A: H_2_O, Eluent B: Acetonitrile) both containing 20 mM formic acid. The gradient began at 10% Eluent B and increased to 100% over 10 min, at which point it was held for 2 min. The UHPLC was coupled to an Agilent 6545 QTOF mass spectrometer equipped with an electrospray ionization source (ESI) with a drying gas temperature of 160°C, a gas flow of 13 l min^−1^, a sheath gas temperature of 300°C, and a flow rate of 16 l min^−1^. Ions were detected in a *m/z* range of 100–1700, in positive ionization mode. Fragmentation was performed on precursors using automated data dependent fragmentation at three collision energies (10, 20, 40 eV) at a rate of 10 spectra s^−1^ and three precursors per cycle. The scan rate was set to 10 spectra s^−1^ in both MS and MS/MS. All were calibrated with 1 μM tributylamine (Sigma-Aldrich) and 10 μM hexakis (2,2,3,3-tetrafluoropropoxy) phosphazene (Apollo Scientific Ltd., Cheshire, UK) as internal lock masses.

The analysis of the resulting dataset was undertaken in a targeted manner, screening all extracts for 16 secondary metabolites (Table S1) known to be produced by roseobacter species. Screening was based on extracted ion chromatograms within an accurate mass of 10 ppm, isotope pattern similarity, and in the case of TDA, an external standard was used to further confirm its identification using retention time and fragmentation pattern alignment. Data analysis was undertaken in Agilent MassHunter version B.07.00 and MassHunter PCDL Manager version B.07.00 build 7024.0.

### Bryozoan microbiome composition analyses

Community DNA for bacterial 16S rRNA gene (V3V4) amplicon sequencing was extracted from bryozoan biomass and from seawater. For each bryozoan sample, 200 mg of biomass was transferred to a Power soil (Qiagen) sample tube and for each water sample (300 ml), one 0.2 μm GE Whatman® polycarbonate filter was transferred to a Power water (Qiagen) sample tube. DNA was subsequently extracted according to the instructions provided by the manufacturer. The purified DNA was used for PCR amplification of the 16S rRNA gene V3V4 region using primers tagged with octameric barcodes [18]. For each PCR reaction, 10.6 μl DNase-free water, 12.5 μl TEMPase Hot Start 2 × Master Mix Blue II (Ampliqon, 290806), 0.32 μm (final conc.) of each primer (Fw_341; 5′CCTACGGGNGGCWGCAG′3, Rv_804; 5′GACTACHVGGGTATCTAATCC′3) [44], and 1 μl DNA template were used. The PCR program consisted of 95°C for 15 min, followed by 30 cycles, with each cycle consisting of 95°C for 30 s, 62°C for 30 s, and 72°C for 30 s, and a final step of 72°C for 5 min. The products were purified using Ampure XP magnetic beads purification (0.6:1 bead volume to DNA solution; Agencourt Bioscience Corporation, Beverly, MA, USA). Concentrations were determined by fluorescence (Qubit™ dsDNA HS assay; Invitrogen by Thermo Fisher Scientific Inc., Eugene, OR, USA) and quality was determined with a DeNovix 439 DS-11+, DeNovix Inc., Wilmington, DE, USA), respectively. Finally, products were pooled in equimolar ratios and sequenced (250PE, Illumina Novaseq 6000) at Novogene (Cambridge, United Kingdom).

Raw reads were demultiplexed, and primer sequences were removed and re-oriented using Cutadapt (v.3.7). Paired-end reads were then processed in R (v.4.2.2) using DADA2 (v.1.28.0), following the procedure described in [45]. ASVs with a length below 380 bases were removed from the dataset. The ASVs were classified using the SILVA database (v.138.1), and contaminants were removed using the “isContaminant” function (method = “prevalence”, threshold = “0.5”) from the decontam package (v. 1.20.0). Subsequent data analyses were conducted in R (v.4.3.1) using phyloseq (v. 1.46.0) for data handling. ASVs with fewer than 10 reads were discarded, resulting in a total of 4739 ASVs. For relative abundance analysis, the ASV table was normalized by percentage normalization. For compositional analysis, the ASV table was normalized using total sum scaling to 100 000 reads per sample and Bray–Curtis dissimilarities were calculated using the “vegdist” function (method = “bray”) from vegan (v. 2.6–4), with a Wisconsin and square root transformed ASV table. Non-metric dimensional scaling (nMDS) ordination was conducted using “metaMDS” (trymax = 1000, k = 2, autotransform = FALSE) from vegan (v. 2.6-4). Permutational Multivariate Analysis of Variance (PERMANOVA) and multivariate homogeneity of group dispersions/variances were determined using the “adonis2” (permutations = 1000) and “betadisper” function, respectively, from vegan (v. 2.6–4). All graphs were generated using ggplot2 (v.3.4.4).

### *tdaC* expression

The expression of the TDA BGC was assessed using reverse transcriptase quantitative PCR (RT-qPCR) targeting the *tdaC* gene, which was found to be the most conserved gene in the TDA BGC. Gene sequences were extracted from the assembled genomes and aligned using CLC main Workbench 8 (CLC bio, Aarhus, Denmark) to produce the final primers (tdaC_jh_fwd; 5′-GGAACGAATTGTGAGCGAG-3′ and tdaC_jh_rev; 5′-GAGTGGAGATGGGGGTAG-3′) using default settings. Sequences were confirmed by BLASTn in CLC main Workbench against the *tdaC* gene. Eight hundred milligram of bryozoan material was collected in Sep, 2021 in 2 ml screw cap micro tubes and immediately snap frozen in a dryshipper with liquid nitrogen. Samples were stored at −80°C. Co-extraction of DNA and RNA was done using RNeasy PowerMicrobiome Kit (Qiagen) following the instructions provided by the manufacturer, with the addition of 100 μl Phenol–chloroform–isoamyl alcohol (25:24:1), pH 8.0 prior to lysis and the use of FastPrep FP120 (Savant) for physical lysis for 30 s at speed 4.5. Turbo DNase was used for DNA removal and the concentrations were measured using Qubit™ RNA HS assays (Invitrogen by Thermo Fisher Scientific Inc., Eugene, OR, USA). Reverse transcription was performed using SuperScript® IV Reverse Transcriptase (Invitrogen) and the tdaC_jh_rev primer. Quantitative PCR amplification was performed on an Opus 96 real-time machine (Bio-Rad Laboratories, Inc., Hercules, CA, USA) in triplicate 20 μl reaction volumes using the Luna Universal qPCR Master Mix (New England Biolabs) and the *tdaC* primers described above in a concentration of 0.25 μm and 1 μl of either DNase treated RNA or 2 μl cDNA. A two-step cycle protocol was employed following the instructions provided by the manufacturer with an annealing temperature of 63°C. Water, no-template, and no-reverse-transcriptase controls were included. Melting curves from all analyses were examined for verification of the right amplification product. A standard curve was from freshly collected bryozoan biomass with the addition of *Phaeobacter* to a concentration ranging from 1.8 × 10^5^ to 1.8 × 10^8^ cells/gram of bryozoan. Prior to DNA extraction, water was removed using a vacuum centrifuge and DNA extracted using the DNeasy PoweSoil Pro (Qiagen, Denmark). All amplicons were verified by Sanger sequencing (Macrogen, Europe).

## Results

Samples were collected from 20 time points across 12 years from April 2009 to May 2021 (Table 1). During the sampling campaigns, seawater temperatures varied from 0.5 to 22°C, while salinity fluctuated in the range of 11–15 ppt. The pH at the sampling site was 8.2 ± 0.22 (determined as the mean from triplicate samples from four sampling time points) and the O_2_ concentration 2.82 ± 0.851 mg L^−1^ (*in situ* measurements at four sampling time points).

**Table 1:** Sampling and *Phaeobacter* isolates obtained from bryozoans in Jyllinge Harbor across a 12-year period from April 2009 to May 2021.

**Sampling time**	**Physico-chemical parameters**				**No. *Phaeobacter* sp. isolates**		**Reference**
	Temp (°C)	Salinity ppt	pH	[O] (mg L^−1^)	Obtained	Sequenced	
April 2009	12	14	ND	ND	0	0	Bernbom *et al.*, 2011
June 2009	16	14	ND	ND	0	0	Bernbom *et al.*, 2011
Aug 2009	19	14	ND	ND	6	6	Bernbom *et al.*, 2011
Oct 2009	7	15	ND	ND	7	7	Gram *et al.*, 2015
Nov 2009	7	15	ND	ND	4	4	Bernbom *et al.*, 2011
Feb 2010	0.5	ND	ND	ND	0	0	Bernbom *et al.*, 2011
June 2011	17	12	ND	ND	0	0	Bernbom *et al.*, 2011
Aug 2011	18	13	ND	ND	11	11	Gram *et al.*, 2015
Sep 2011	15	11	ND	ND	0	0	Gram *et al.*, 2015
Aug 2012	20	14	ND	ND	4	4	Gram *et al.*, 2015
Sep 2012	16	14	ND	ND	14	14	Gram *et al.*, 2015
Aug 2014	ND	ND	ND	ND	13	9	This study
Sep 2018	16	15	ND	2.27	19	19	This study
Oct 2019	9	14	7.98	2.48	0	0	This study
July 2020	17	14	ND	ND	9	8	This study
Aug 2020^a^	22	15	8.51	2.23	23	21	This study
Sep 2020	21	14	8.26	4.28	11	11	This study
May 2021	13	ND	8.18	ND	0	0	This study
July 2021^b^	ND	ND	ND	ND	ND	ND	This study
Sep 2021^b^	ND	ND	ND	ND	ND	ND	This study
**Total**					**121**	**112**	

a Two sampling time points separated by 11 days.

b Only material for DNA and RNA analyses were collected on these dates.

ND: Not determined.

**Figure 1:**
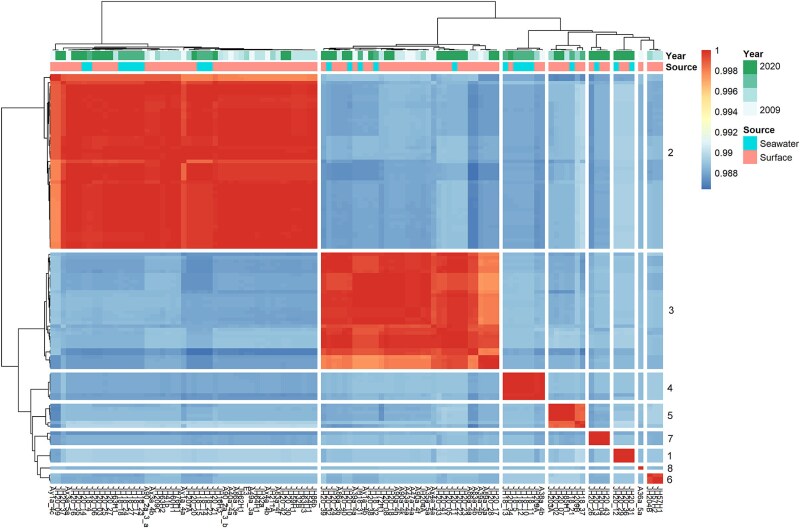
Genomic heterogeneity among the 112 *Phaeobacter* sp. genomes depicted as the average nucleotide identity (ANI) on a percentage scale in decimal representation. ANIs among the genomes were above the species threshold (95%) for all genomes yet eight distinct clades (indicated by numbers 1–8 on the right side of the heatmap) were observed within the species. The distribution of the clades among different isolation sources and years did not indicate succession patterns or the presence of specific ecotypes.

A total of 121 *Phaeobacter* isolates were obtained from 11 of the 19 samplings, being absent from samples collected in April and June 2009, February 2010, June 2011, September 2011, October 2019, and May 2021, suggesting that the *Phaeobacter* population oscillated in abundance between summer/autumn and winter/spring (Table 1). Whole-genome sequencing of 112 of the 121 isolates showed that the population represented a single species with a minimum Average Nucleotide Identity (ANI) between isolates of 0.986. Furthermore, the population comprised eight clearly distinguishable clades, which represented between one and 52 individual isolates (Fig. 1). To assign the isolates to any known species of *Phaeobacter*, the genomes were compared to four closely related reference species, *P. inhibens* 2.10, *P. inhibens* DSM 16374^T^, *P. inhibens* DSM 17395, and *Phaeobacter piscinae* DSM 103509^T^. The isolates from Jyllinge Harbor all exhibited ANIs to the reference strains in the range of 0.86–0.87, suggesting that these isolates did not represent any of the reference species (Fig. S1). This was further supported by a phylogenomic comparison with the strains in the Type strain Genome Server (TYGS), which placed representatives of each of the eight clades as one unique species distinct from any known type strain (Fig. S2). Further phenotypic testing corroborated the assignment to a novel species (*Candidatus* Phaeobacter bryozoorum) and are presented elsewhere [46].

**Figure 2:**
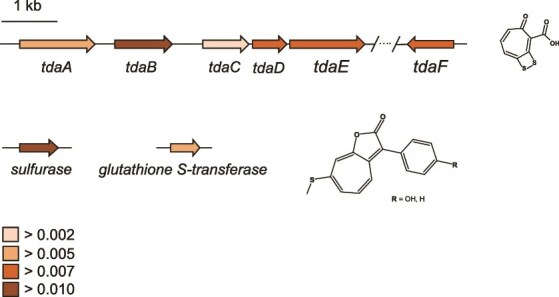
The diversity in biosynthetic genes (entropy at the amino acid level) involved in TDA production and roseobacticide production in *Phaeobacter* sp. among members of the eight clades. All 112 isolates harbored a complete TDA BGC as well as genes encoding the sulfurase and glutathione S-transferase genes unique to roseobacticide producing *Phaeobacter* spp.

To delineate any potential ecological differentiation between clades within the novel *Phaeobacter* species, we investigated if the source material (seawater or surface attached bryozoan community) could be linked to individual clades. This was not the case as individual isolates obtained from seawater were scattered among the isolates obtained from surface attached communities (Fig. 1). All isolates harbored a complete TDA BGC as well as the two additional genes encoding a glutathione S-transferase and a sulfurase (Fig. 2), which distinguishes roseobacticide producing roseobacter species from nonproducing species [47]. As isolates appeared highly similar in their genomes, and as they appeared to be identical ecotypes based on their niche distribution, we used a novel approach to ascertain to what extent TDA cluster gene sequences varied among clades and to what extent the capacity to produce TDA is conserved across a decade. By calculating the average entropy of both TDA cluster genes and genes potentially associated with roseobacticide production, we found the biosynthetic genes to be highly conserved among all isolates, exhibiting entropies between 0.0021 and 0.011, with *tdaC* being most conserved and *tdaB* being the least conserved gene in the TDA cluster. For reference, the housekeeping genes *gyrA, rpoB,* and *recA* are almost completely conserved, i.e. have entropies of 0.001, 0.0002, and 0, respectively, which is significantly lower than the TDA cluster (*P <* .001). Hence, TDA and likely also roseobacticide production, are conserved traits in the *Phaeobacter* sp. population and therefore likely central to their ecology. Collectively, these data show that all eight clades indeed represent a single ecotype present in both seawater and surface attached bryozoan communities, and that they in general have the capacity to antagonize specific bacterial community members and lyse algal cells. Targeted metabolomic analysis corroborated the presence of TDA as well as the TDA analog methyl-troposulfenin [48] across all strains (Fig. S3). Also, all strains were capable of producing the quorum sensing molecule 3-hydroxydecanoylhomoserine lactone (3OH-C10-HSL), which is involved in coordinated gene production of algicidal compounds in *P. inhibens*. The relative abundances of these features were similar across all strains, with the highest relative abundances of TDA in the strains isolated from Jyllinge Harbor compared to the reference species.

**Figure 3:**
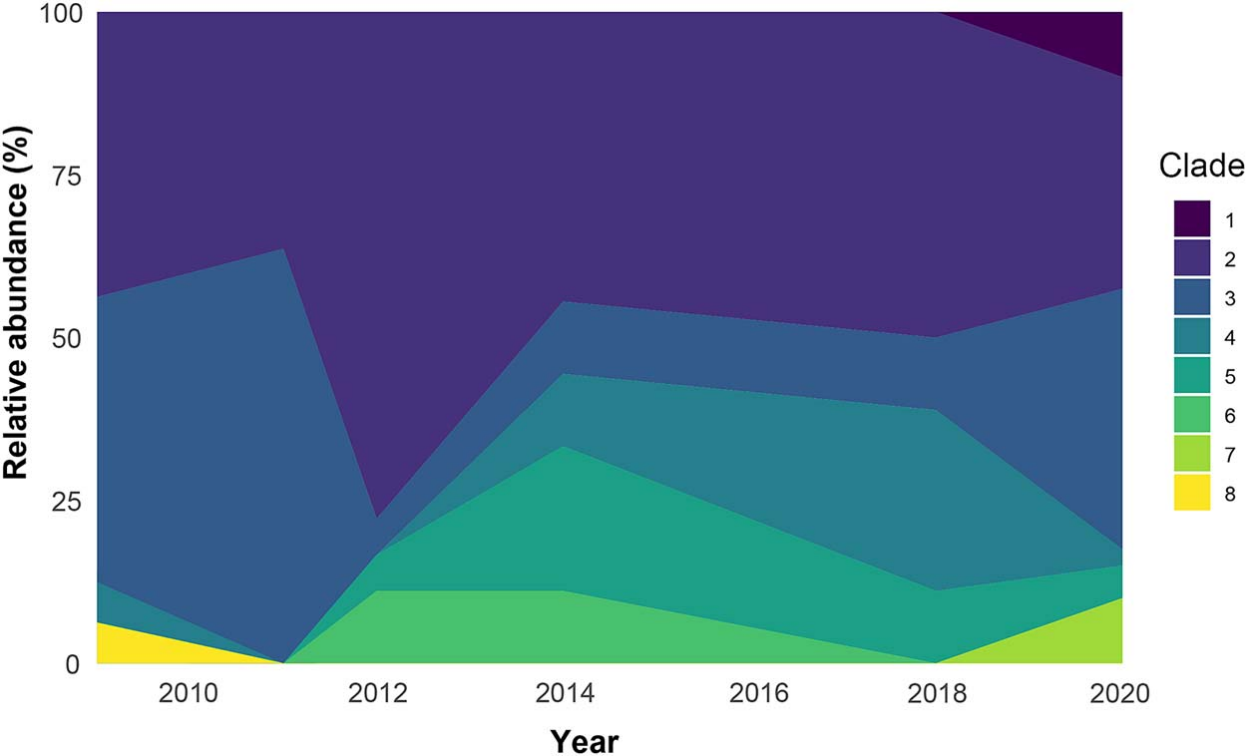
Relative abundance of the eight *Phaeobacter* clades from August 2009 to September 2020 based on cultivation and whole-genome sequencing.

To uncover any potential indications of succession by individual clades over time, we investigated temporal patterns in relative clade abundance among isolates obtained from different years (Fig. 3). The two largest clades, Clade 2 and 3, were recovered at all sampling time points where *Phaeobacter* sp. was observed and comprised 33%–78% and 5.6%–64% of the relative abundance throughout the period from August 2009 to September 2020, respectively. Three less abundant clades, Clade 1 (four isolates), Clade 7 (four isolates), and Clade 8 (one isolate) were only observed once, whereas the remaining three clades persisted for at least two consecutive seasons (Fig. 1, Fig. 3). Thus, as some individual clades persist in the system for more than a decade, the population is not likely to be re-established yearly from allochthonous sources, but is more likely oscillating autochthonously, albeit at very low abundances between seasons.

**Figure 4:**
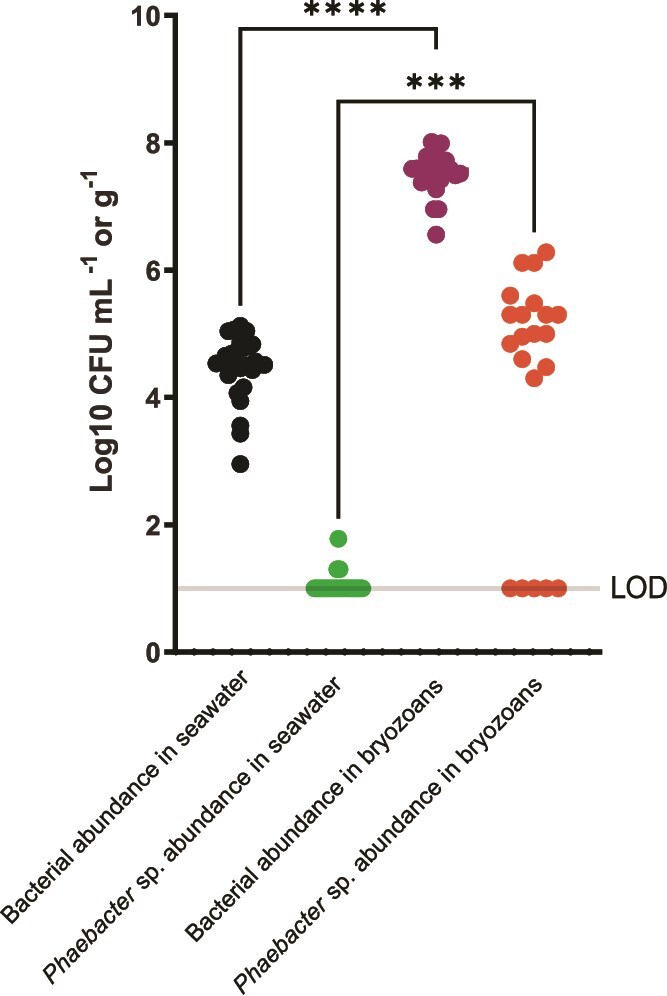
Absolute abundance (CFUs) of cultivable bacteria and *Phaeobacter* sp. in seawater and in bryozoans of Jyllinge harbour from Sep 2018 to Sep 2020. LOD: limit of detection (1 Log_10_ CFU ml^−1^ or g^−1^, or 1 CFU in 100 μl inoculum).

**Figure 5:**
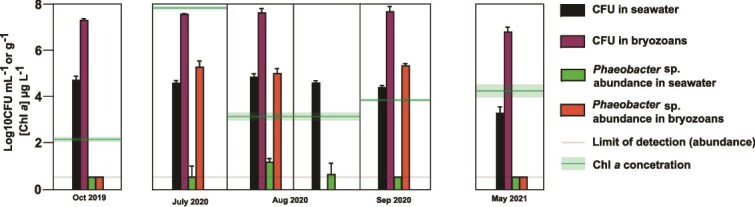
Absolute abundance (CFUs) of cultivable bacteria, *Phaeobacter* sp., and chlorophyll *a* (Chl *a*) concentrations in seawater and byrozoans in Jyllinge harbour. Note that only seawater was sampled at the second sampling time point in August.

To confirm the oscillation pattern, we investigated population dynamics in more detail across a two-year period (September 2018–September 2020) by determining absolute abundances of *Phaeobacter* sp. in the system. The abundances exhibited a bi-modal distribution during this period, where it was either present at high abundances or below the detection limit. This pattern was not observed for the cultivable fraction of bacteria in the system in general (Fig. 4). The bi-modal distribution was observable both in seawater, where *Phaeobacter* sp. abundances did not exceed 1.8 Log_10_ CFU ml^−1^, and in the surface attached bryozoan-associated communities, which by contrast exhibited significantly higher *Phaeobacter* sp. abundances with a mean of 4.7 Log_10_ CFU g^−1^ (Dunn’s test, *P* = .0002). Increasing resolution around the summer of 2020 showed that *Phaeobacter* sp. abundances in seawater, and in association with bryozoan communities, increased in the summer months averaging 0.89 Log_10_ CFU ml^−1^ ± 0.74 Log_10_ CFU ml^−1^ (s.d.) in seawater and 5.2 Log_10_ CFU g^−1^ ± 0.23 Log_10_ CFU g^−1^ (s.d.) in bryozoans, whereas they were below the detection limit in the preceding October samples from 2019 and the succeeding spring samples (May 2021; Fig. 5; Table S2). The abundance peaked in July 2020 but was generally high throughout the summer months within bryozoans whereas abundances in seawater were shifted relative to the bryozoan associated population, peaking in August (1.2 Log10 CFU ml^−1^ ± 0.14 Log_10_ CFU ml^−1^ (s.d.)). Hence, the population of *Phaeobacter* sp. was already established in the bryozoans prior to the peak in seawater, suggesting that the seawater population may be seeded from the bryozoan-associated *Phaeobacter* sp. population.

**Figure 6:**
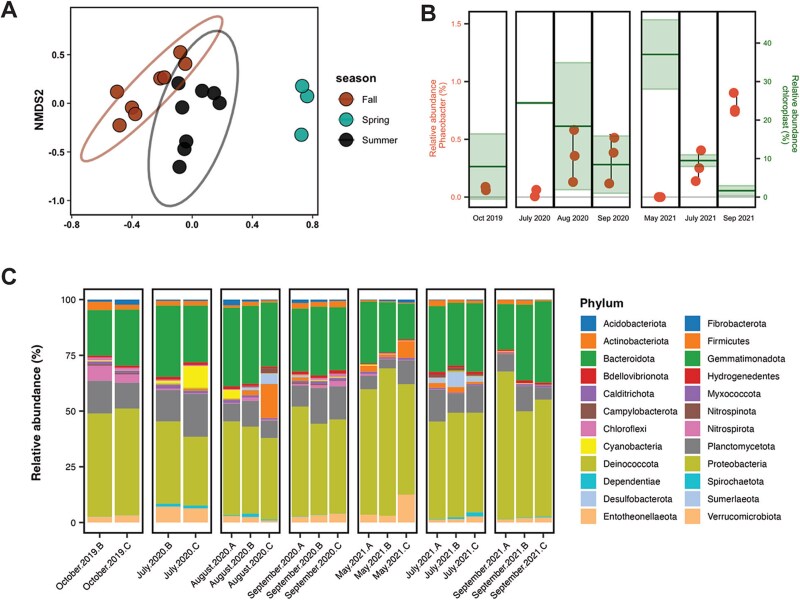
Bryozoan microbiomes (bacterial 16S rRNA gene (V3V4) amplicon sequencing). (A) Microbial community compositions visualized as Bray–Curtis distances in a nMDS ordination, colored according to season (spring: May, summer: July–August, fall: September–October; stress = 0.125). (B) Relative abundance (%) of *Phaeobacter* sp. (ASV_135) in the bacterial community (orange). Each point represents replicates, and black vertical lines show standard deviations. Horizontal green line represents mean chloroplast relative abundance (%) of all 16S rDNA V3V4 sequences (secondary y-axis). Shaded green shows standard deviation. (C) Relative abundance of bacterial phyla assigned in the 16S rDNA V3V4 sequences. Each sample is denoted by sampling month, year, and replicate (A, B, or C).

To further elucidate population dynamics in the bryozoan microbiome and determine if there was a relation between *Phaeobacter* sp. and algal abundance (relative chloroplast abundance) within the bryozoans, we analyzed community composition through 16S rRNA gene V3V4 amplicon sequencing on samples collected from October 2019 to September 2021. Looking at the bacterial community structure, samples clustered according to season (PERMANOVA; *P* < .001), where the difference in microbial community composition between the spring and summer–fall was most distinct (Fig. 6A). One ASV (ASV135) was identified as belonging to the genus *Phaeobacter* and was identical to the 16S rRNA gene sequence obtained from the genomes of the isolated *Phaeobacter* sp. This ASV exhibited an oscillating pattern in relative abundance that mirrored the pattern observed in the cultivable counts of the *Phaeobacter* sp., suggesting that other phaeobacters were not present in the bryozoans. Its relative abundance ranged from 0.8 ± 0.1% in September 2021 to undetectable levels in May 2021 (Fig. 6B, Table S2). The notable exception to this pattern occurred in July 2020. During this period, the relative abundance of the *Phaeobacter* ASV in bryozoans was recorded at 0.04 ± 0.04%. In contrast, the cultivable counts of *Phaeobacter* were among the highest recorded in July 2020, as previously mentioned (Fig. 5A), suggesting that other taxa (i.e. algae and cyanobacteria) were increasing in abundance relative to *Phaeobacter* within the bryozoans at this time (Fig. 6C). The highest relative chloroplast abundances were observed in May 2021 (37.0 ± 8.9%) and July 2020 (24.4 ± 0.24%) and coincided with the highest seawater Chl *a* concentrations (4.29 ± 0.3 μg L^−1^ and 7.84 ± 0.1 μg L^−1^, respectively; Fig. 5A). In May 2021, the *Phaeobacter* ASV could not be detected in the bacterial community and in general the relative *Phaeobacter* abundances in bryozoans were offset relative to chloroplast abundances. Collectively, the absolute and relative abundances of *Phaeobacter* suggested that the population primarily resides within bryozoans and are not accumulating passively due to filter feeding by the bryozoan on a primary algal host. Rather, *Phaeobacter* sp. appears to accumulate and persist within its bryozoan host as algal biomass is turned over. To substantiate an active association with the bryozoan host further, we confirmed that *Phaeobacter* sp. was actively producing TDA (*tdaC* expression) in association with the bryozoans in September 2021 where its relative abundance was at its maximum observed (0.8 ± 0.1%) and where relative chloroplast abundances within the bryozoans had diminished after the summer peak (Fig. 6B). Here, we observed a mean *tdaC* transcript abundance of 1.4 × 10^4^ transcripts g^−1^ in triplicate samples.

## Discussion

The roseobacter genus, *Phaeobacter*, occupies several marine host-associated niches [10, 26], and their intricate bifunctional association with microalgae, where *Phaeobacter* both acts as a mutualistic symbiont and a parasite [30], underscores their importance in marine carbon sequestration and remineralization. Our results show that *Phaeobacter* occupies an additional niche as part of the microbiome of the encrusted bryozoan genus *Conopeum*, where it recurs and thrives at high abundances in the late summer and early autumn months.

The *Phaeobacter* sp. isolates obtained from bryozoans and seawater across a decade in the estuarine harbor environment all represented a single novel species [46], suggesting that it is highly adapted to the niche in which it occurs, and thus competitively excludes other *Phaeobacter* spp. [18, 49]. Specifically, it was observed at high relative abundances and with high cultivable counts in association with bryozoans. *tdaC* amplicons from putative *Phaeobacter* spp. have been obtained from other marine animals, particularly the amphipod *Protophylae niger* [26], which is a prominent grazer of the *Phaeobacter* host alga *Ulva* [50–52]. Hence it can be difficult to delineate to what extent *Phaeobacter* is an integral part of the animal microbiome or whether it accumulates here as a result of ingestion of algae with which *Phaeobacter* associates. The infrequent observation of *Phaeobacter* in seawater observed in this study suggests that while roseobacters may have high relative abundances in coastal waters [53], where they are among the primary glucose consumers [54], the species at hand predominantly resides in association with its bryozoan host and not with phytoplankton. The Roskilde Fjord estuary, in which the harbor is situated, experiences high loads of allochthonous inorganic nutrients supporting significant algal blooms [55, 56] and consequently high amounts of bioavailable dissolved organic matter [57], which could favor heterotrophic algae-associated roseobacters, such as *Phaeobacter*. However, investigations of microbial community function and composition in the system across a yearly cycle corroborates the low abundances observed in seawater in the present study [58]. Moreover, the population was already established within bryozoans, prior to the point where *Phaeobacter* was detectable in seawater. Hence, population dynamics combined with the fact that we observed active transcription of the *tdaC* gene in the bryozoan host implies that *Phaeobacter* is an active and integral part of the microbiome and does not accumulate directly due to ingestion of a primary algal host.

All sequenced isolates harbored a complete TDA BGC, and moreover they harbored the sulfurase and glutathione S-transferase genes proposed to separate roseobacticide producers from nonproducers in the *Phaeobacter* genus [47]. Metabolomic analyses of the isolates confirmed that *Phaeobacter* sp. is a proficient producer of TDA and previous analyses of select representatives of the species demonstrated their ability to produce roseobacticides upon algal cues [34, 47] as well. We observed a high degree of conservation of the TDA genes across a decade in the population and we believe that these secondary metabolites are essential to their ecological role in the bryozoan microbiome. While the approach used in our study selects for TDA producers (isolation based on colony color on MA), we only identified one *Phaeobacter* ASV in the amplicon data and this was 100% identical to the isolated strains, corroborating that only this TDA-producing ecotype was present in the system. Despite the antibacterial properties of TDA, we have previously demonstrated that the presence of TDA producers on a surface may attract several genera and the ability to produce TDA may even render *Phaeobacter* a keystone species in marine microbiomes [59]. Despite this, it is still widely assumed that the antimicrobial effect of TDA plays a significant role in the protection of its eukaryotic host [30], why *Phaeobacter* has also been proposed as a potent aquaculture probiotic [60–62]. As the bryozoan immune system solely consists of the innate branch, it is likely that these invertebrate animals rely on their associated microbiome to produce antimicrobial secondary metabolites. We observed expression of *tdaC* within the bryozoan, suggesting that *Phaeobacter* sp. produce TDA in the host and hence may protect it from invading pathogens. Bioactive secondary metabolites, i.e. bryostatins, have been isolated from bryozoans previously [63] and have later been attributed to its associated bacterial obligate symbiont *Endobugula sertula* [64]. To what extent bryostatins aid in host defense is however, not currently known. Host-protective microbial secondary metabolites have been described in more detail for marine sponges, which are among the oldest metazoans and host diverse microbiomes encoding extensive repertoires of bioactive secondary metabolites [65]. Species of the yet-uncultured sponge-associated *Enthotheonella* genus are particular rich in BGCs and interestingly, we also observed ASVs of *Enthotheonellota* in association with the bryozoans in Jyllinge harbor, albeit at low abundances. The fact that representative isolates of the bryozoan-associated *Phaeobacter* sp. produce roseobacticides upon exposure to the algal degradation product *p*-coumaric acid [66] further indicates that it may play an important role in the bryozoan microbiome by aiding in lysis and digestion of phytoplankton biomass. The *Phaeobacter* population exhibited an oscillating abundance pattern that contrasted with the total cultivable part of bryozoan-associated community. *Canopeum* growth rates are generally low in winter due to decreased temperatures and reduced concentrations of phytoplankton and particulate organic matter [67]. We speculate that during this period, the bryozoan host becomes largely inactive, limiting nutrient availability to its associated microbiome. As a result, *Phaeobacter* populations likely decline to a subdetection levels until the bryozoan host resumes active feeding and organic matter intake, allowing the population to re-establish.

In conclusion, we show that in addition to their dual interactions with haptophytes, TDA and roseobacticide producing phaeobacters, here exemplified by the novel species *Candidatus* Phaeobacter bryozoorum, are an active part of the bryozoan microbiome. Comparison of more than a hundred genomes of the species collected across a decade showed that they are highly adapted to the niche and persist as a homogenous population across decades despite oscillating in abundance in accord with the activity of the filter feeding host organism.

## Supplementary Material

Supplemental_material_ycaf178

## Data Availability

The datasets generated during and/or analysed during the current study are available, for WGS of *Phaeobacter* sp. isolates, on NCBI under PRJNA1061128 (www.ncbi.nlm.nih.gov/bioproject/PRJNA1061128/), and for V3V4 16S amplicon sequencing reads, in the SRA database at NCBI under BioProject no. PRJNA1277747 (www.ncbi.nlm.nih.gov/bioproject/PRJNA1277747/).
