# Supplementary material for: Autecology of an oscillating population of a novel host-associated Phaeobacter species proliferating in marine bryozoans
Source: ISME Commun. 2025 Oct 9;5(1):ycaf178. doi: 10.1093/ismeco/ycaf178 (PMC12596164; doi:10.1093/ismeco/ycaf178)
Supplement: Supplemental_material_ycaf178 [file supplemental_material_ycaf178.pdf]

Supplemental Material for

The autecology of an oscillating population of a novel host-associated *Phaeobacter* species proliferating in marine bryozoans

Mikkel Bentzon-Tilia, Nathalie N. S. E. Henriksen, Morten D. Schostag, Aaron J. C. Andersen, Jette Melchiorsen, Mikael L. Strube, Lone Gram

Technical University of Denmark, Department of Biotechnology and Biomedicine, Søltofts Plads Bldg. 221, 2800 Kgs. Lyngby

Address correspondence to Mikkel Bentzon-Tilia: mibti@dtu.dk

Running title: The autecology of a host-associated Phaeobacter species

Keywords: *Phaeobacter*, roseobacters, tropodithietic acid, TDA, roseobacticides, bryozoans

Table S1. Target list of secondary metabolites for targeted dereplication by LC-MS.

Name	Molecular Formula
**Methyl-troposulfenin**	$C_9H_6O_3S_2$
**R-3OH-C10-HSL**	$C_{14}H_{25}NO_4$
**Roseobacticide A**	$C_{16}H_{12}O_3S$
**Roseobacticide B**	$C_{16}H_{12}O_2S$
**Roseobacticide C**	$C_{18}H_{13}NO_2S$
**Roseobacticide D**	$C_{16}H_{12}O_3S_2$
**Roseobacticide E**	$C_{16}H_{12}O_2S_2$
**Roseobacticide F**	$C_{18}H_{13}NO_2S_2$
**Roseobacticide G**	$C_{15}H_{10}O_5S$
**Roseobacticide H**	$C_{21}H_{14}O_4S$
**Roseobacticide I**	$C_{21}H_{14}O_3S$
**Roseobacticide J**	$C_{30}H_{18}O_4S_2$
**Roseobacticide K**	$C_{30}H_{18}O_5S_2$
**Roseochelin A**	$C_{21}H_{20}O_8$
**Roseochelin B**	$C_{21}H_{20}O_8S$
**Tropodithietic acid**	$C_8H_4O_3S_2$

Table S2. Detection and abundance of *Phaeobacter* sp. across sampling time points and methods.

	**Detection and abundance of *Phaeobacter* sp.**		
**Sampling time**	Isolation (+/-)	Mean absolute abundance in bryozoans (log_{10} CFU g^{-1})	Mean relative abundance in bryozoans (%)
April 2009	-	ND	ND
June 2009	-	ND	ND
Aug 2009	+	ND	ND
Oct 2009	+	ND	ND
Nov 2009	+	ND	ND
Feb 2010	-	ND	ND
June 2011	-	ND	ND
Aug 2011	+	ND	ND
Sep 2011	-	ND	ND
Aug 2012	+	ND	ND
Sep 2012	+	ND	ND
Aug 2014	+	ND	ND
Sep 2018	+	1.67	ND
Oct 2019	-	0[c]	0.04
July 2020	+	5.30	0.06
Aug 2020[a]	+	5.03	0.2
Sep 2020	+	5.36	0.3
May 2021	-	0[c]	0
July 2021[b]	ND	ND	0.2
Sep 2021[b]	ND	ND	0.8

[a]Two sampling time points separated by 11 days.

[b] Only material for DNA and RNA analyses were collected on these dates.

[c] Below LOD (1 Log10 CFU mL-1 or g-1, or 1 CFU in 100 µL inoculum)
ND: Not determined

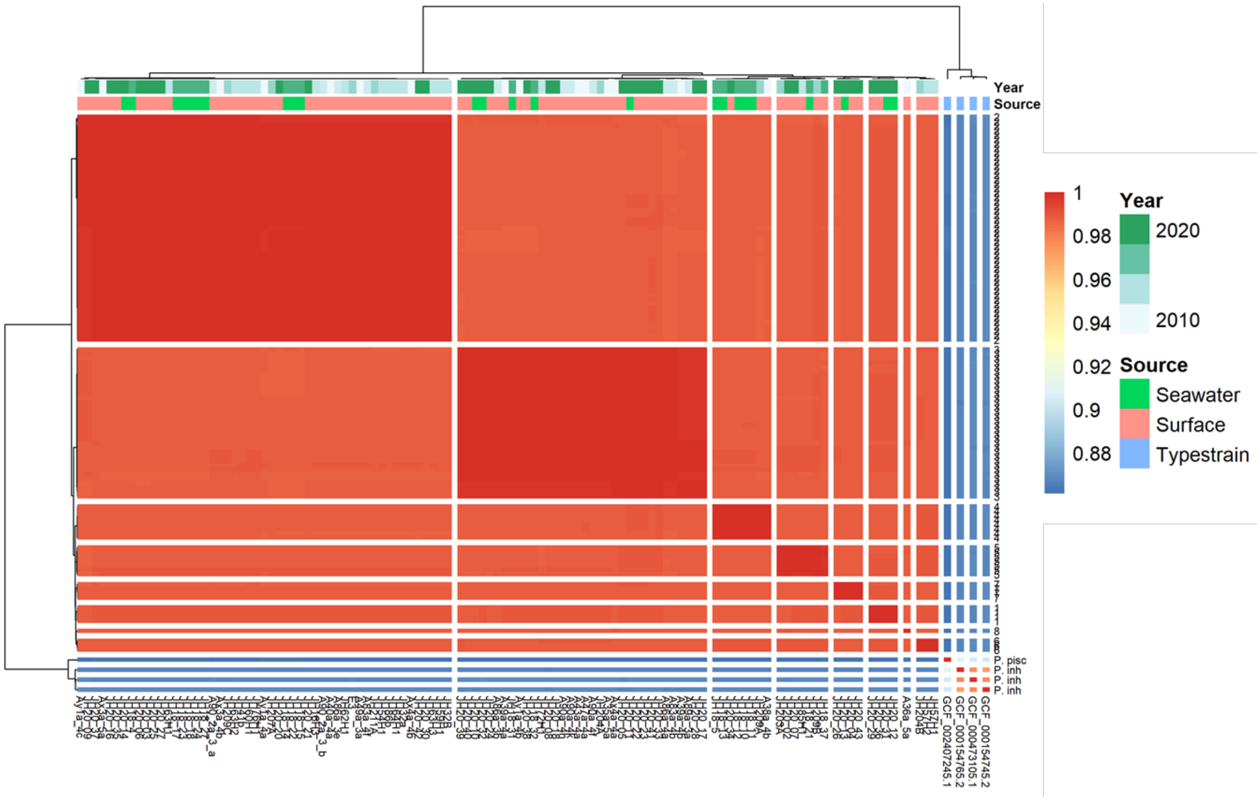

Figure S1. Genomic heterogeneity among the 112 *Phaeobacter* sp. genomes depicted as the average nucleotide identity (ANI) including the closely related reference strains *P. inhibens* 2.10, *P. inhibens* DSM 16374, and *P. piscinae* P14.

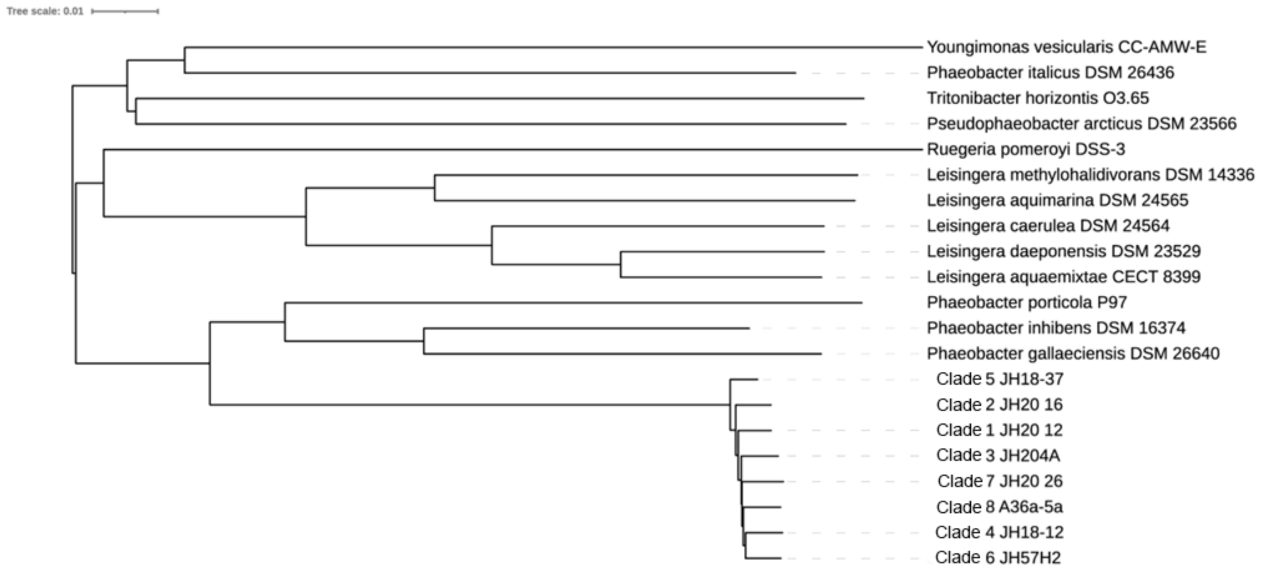

Figure S2. Phylogenomic tree produced using the TYGS including one representative genome from each of the eight clades, corroborating that the *Phaeobacter* sp. isolates from Jyllinge Harbor represent a new species.

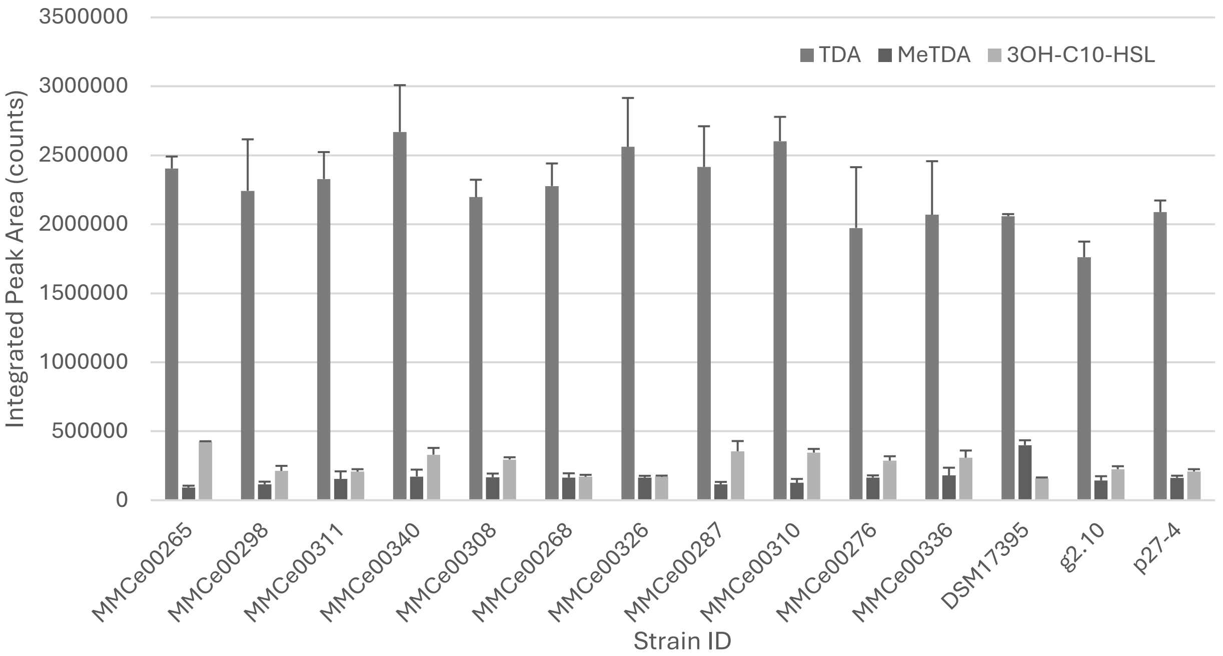

Figure S2. Integrated peak areas from targeted metabolomic analyses of 11 selected strains of *Phaeobacter* sp. (MMCe00265-MMCe00340) and reference strains *P. inhibens* DSM17395, *P. inhibens* 2.10, and P. piscinae DSM 103509^{T} (27-4). TDA: tropodithietic acid, MeTDA: Methyl-troposulfenin, R-3OH-C10-HSL: 3-hydroxydecanoylhomoserine lactone.
